# Fillings as Barriers to Bacteria

**Published:** 1903-10-15

**Authors:** A. E. Webster

**Affiliations:** Toronto, Can.


### FILLINGS AS BARRIERS TO BACTERIA.

BY A. E. WEBSTER, TORONTO, CAN.

The author has carried on a series of investigations to determine the degree of efficiency of temporary and permanent filling materials as barriers to bacteria. From this work he has been able to draw some interesting conclusions, namely, that gutta-percha, temporary stopping, cement, cotton and sandarac, cotton, vaselin and cement, or gutta-percha, are of no value for sealing a cavity in a tooth to prevent infection from the oral cavity. As a result of his researches he feels safe in stating that zinc oxyphosphate cements, gutta-percha, cotton, or any combination of these with each other or with any other non-disinfecting materials will not prevent the passage of moisture or bacteria. Zinc oxychlorid will prevent the passage of bacteria for at least sixty days, but not of moisture for that length of time. Some amalgams, if properly mixed and inserted, will resist the passage of bacteria for two months, while others, no matter how mixed or inserted, will not resist them for three hours.

Regarding cements, Dr. Webster states that he has tested ten or twelve cements of different names, and that his conclusion is that they all leak. Those that do not shrink allow the passage of moisture and bacteria through the mass, the only difference being that those that shrink leak sooner than those that do not. He has tested only a few amalgams, and has reached the conclusion that those that contract or flow badly are quite likely the only ones that leak. Root-canal fillings made by heating gutta-percha cones and packing them with instruments that penetrate the material are not so resistant to either moisture or bacteria as those made by first using chloro-percha and then carefully forcing the proper-sized cone into the canal without puncturing or heating. When gutta-percha is heated it seems to acquire a tendency to creep, and, unless it is held with continuous pressure until it is hard, spaces will appear around it. Notwithstanding its defects, chloro-percha root fillings are the best resistants to moisture among those materials examined, but this resistance is not great enough to make it an ideal filling.

His final conclusion is that few, if any, root-canals fillings are resistant to either moisture or bacteria, and that most peridental infections are direct from the oral cavity in pulpless teeth, and were zinc oxychlorid used in the pulp chamber or some part of the root-canal, many if not all such infections would be averted.—*Cosmos. Extract from Dental Review.*

